# New insights into the mechanism of low high-density lipoprotein cholesterol in obesity

**DOI:** 10.1186/1476-511X-10-176

**Published:** 2011-10-12

**Authors:** Hao Wang, Dao-Quan Peng

**Affiliations:** 1 Departments of Cardiology, the Second Xiangya Hospital, Central South University, Changsha, 410011, Hunan, P.R. China

**Keywords:** Obesity, HDL-C, adipocytes, hepatocytes, microRNA-33

## Abstract

Obesity, a significant risk factor for various chronic diseases, is universally related to dyslipidemia mainly represented by decreasing high-density lipoprotein cholesterol (HDL-C), which plays an indispensible role in development of cardiovascular disease (CVD). However, the mechanisms underlying obesity and low HDL-C have not been fully elucidated. Previous studies have focused on the alteration of HDL catabolism in circulation following elevated triglyceride (TG). But recent findings suggested that liver and fat tissue played pivotal role in obesity related low HDL-C. Some new molecular pathways like microRNA have also been proposed in the regulation of HDL metabolism in obesity. This article will review recent advances in understanding of the potential mechanism of low HDL-C in obesity.

## Introduction

Following the change of dietary structure and the limitation of physical activity, the increasing prevalence of obesity is becoming a serious threat to global public health. Obesity is associated with various chronic diseases, particularly cardiovascular diseases (CVD), diabetes mellitus type 2, sleep apnea, certain types of cancer and osteoarthritis [1]. In humans, one of the characteristics of obesity is dyslipidemia which includes high levels of triglycerides (TG) in very-low-density lipoproteins (VLDL) and low levels of high-density lipoprotein cholesterol (HDL-C) [2]. Actually, dyslipidemia is a more valuable predictor for the development of CVD compared to other manifestations of obesity. Findings of the Emerging Risk Factors Collaboration's study demonstrated that assessments of body size could not compensate for the lack of blood lipids assay, particularly the information about total cholesterol and HDL-C [3]. It is widely known that low plasma HDL-C levels are inversely related to the risk of CVD and independent of other risk factors [4,5]. In addition, under the state of obesity, HDL particles can be modified to generate dysfunctional HDL which is indicated as a more important risk factor for CVD [6]. The degeneration of HDL that typically accompanies obesity is therefore of considerable importance, whereas the mechanisms underlying obesity and HDL alteration have not been fully elucidated. In this review, we are going to introduce the changes of HDL metabolism in obese individuals and focus on the new insights of the potential mechanism.

## Obesity-related changes in HDL metabolism

### Metabolism and function of HDL

HDL plays a critical role in cholesterol homeostasis. It mediates the transfer of cholesterol from extra hepatic tissues to the liver. This process of reverse cholesterol transport (RCT) is generally thought to be the central antiatherogenic effect of HDL [7]. There are several steps involved in the RCT. To begin with, lipid-poor apoA-I (the major apolipoprotein of HDL) is secreted from the liver or intestine and released into the plasma for circulation to peripheral cells, where it removes excess cholesterol, forming nascent HDL. In the first step of RCT, a specific cell membrane protein, ATP-binding cassette transporter A1 (ABCA1) plays a key role in cellular cholesterol efflux [8]. The ABCA1 shuttles back and forth, transferring cholesterol from lipid pool to apoA-I. Then the free cholesterol on HDL surface is esterified by lecithin cholesterol acyl transferase (LCAT) [9]. Next, the cholesterol esters move to the HDL core, forming the more spherical mature HDL_3_. HDL_3 _can further promote cellular cholesterol efflux through ATP-binding cassette transporter G1 (ABCG1) and scavenger receptor class B type I (SR-BI) [10]. As the HDL_3 _gets more cholesterol, it expands to HDL_2_. Now rich in cholesteryl ester (CE), HDL_2 _engages in the exchange with TG-rich lipoproteins mediated by cholesteryl ester transfer protein (CETP) [11]. The remaining HDL_2 _returns to the liver, interacting with SR-BI receptor which removes cholesterol and converts HDL_2 _to HDL_3_. Liver cholesterol can then be reutilized in VLDL assembly or transformed into bile acids [12]. Through RCT process, peripheral tissues, like vessel-wall macrophages, remove their excess cholesterol, thus preventing cholesterol accumulation and atherosclerotic plaque formation. Furthermore, HDL also exerts other antiatherogenic and vascular protective functions such as antioxidative, antithrombotic and anti-inflammatory actions [7]. Epidemiologic studies have shown that there was an inverse correlation between HDL-C levels and the risk of CVD. For a 5 mg/dl decrement in HDL-C between individuals, there was a 14% incremental risk of cardiovascular events [13]. Each 1 mg/dl increase in HDL-C is associated with a 2%-3% reduction in the risk of CVD [14]. As a result, HDL-raising therapies are considered to be a promising way to reduce the morbidity of CVD. However, a number of factors have been shown to contribute to the reduction of HDL-C. In particular, obesity is the most frequent cause of low concentration of HDL-C and HDL dysfunction.

### Obesity affects the quantity of HDL

There are a great number of evidences that the concentrations of HDL-C are adversely altered in obese people, especially in the case of metabolic syndrome. Obesity is the fundamental manifestation of metabolic syndrome which also includes insulin resistance, hypertriglyceridemia, reduced HDL-C level, elevated blood pressure and glucose intolerance. Metabolic syndrome is highly prevalent. Datas from the Third National Health and Nutrition Examination Survey showed that the age-adjusted prevalence of metabolic syndrome was 23.7% [15]. The most common component of the metabolic syndrome was obesity (39%) followed immediately by the low HDL-C levels (37%) [16].

Generally speaking, HDL-C levels are associated with both the degree and distribution of obesity. Lipid Research Clinics Program Prevalence Study for 6865 white people suggested that the Quetelet index of body mass was significantly and inversely associated with plasma HDL-C in children and adults of both sexes. The relationship between body mass and HDL-C was independent of smoking, alcohol intake, exogenous estrogen hormone use, and TG levels [17]. In the third examination cycle of the Framingham Offspring Study for 1566 men and 1627 women, BMI was inversely and linearly associated with HDL-C concentration. As the BMI increased, there was a steady increase in age-adjusted levels of TG and a decline in HDL-C [18]. Specifically, the correlation of low HDL-C with obesity seems to be strongest with central obesity which is characterized by intra-abdominal or visceral fat deposition. Compared to mid-thigh fat, intra-abdominal fat was a more critical independent predictor for low HDL_2 _levels in obese premenopausal women [19]. The level of HDL-C was inversely correlated with abdominal circumference which directly represents the degree of central obesity and better predicts of low HDL-C levels [20]. Increasing intra-abdominal fat area, quantified by computed tomography scan, was an important determinant of decreased HDL-C [21].

### Obesity affects the quality of HDL

Obesity not only affects the concentration of HDL-C in plasma, but also has an influence on the functionality of HDL. Emerging evidence indicates that HDL can lose its protective activity and even become atherogenic under certain conditions. The dysfunctional of HDL have been shown to be associated with complications of obesity, such as infection, inflammation, diabetes and CVD [22].

Compared to normal HDL, HDL from patients in an acute phase reaction did not inhibit monocyte chemotaxis but actually increased it [23], demonstrating the anti-inflammatory function is impaired. Furthermore, The anti-oxidant function of HDL cholesterol is significantly altered in obese patients compared with healthy controls and HDL isolated from type 2 diabetic patients has displayed low endothelial protective activities [24]. Sasahara et al [25] studied the pathway of cholesterol efflux from fibroblasts by testing plasma samples from obese and lean subjects. Compared to lean individuals, the overall capacity of HDL to promote cholesterol efflux is reduced in obese ones. Considering cholesterol efflux capacity, independently of the HDL-C level, is the main metric of HDL function and has a strong inverse association with coronary artery disease [26], the impaired cholesterol efflux capacity may have a more crucial impact on the development of CVD in obese individuals.

Even though it is undeniable that obesity influences the metablism of HDL, there are some disputes on the explanations for that phenomenon. Up to now, several kinetics studies put forward some hypothesis that factors both intrinsic to HDL particles and extrinsic to HDL particles were involved in obesity related HDL-C reduction. One hand, the overproduction of free fatty acid(FFA) and VLDL is regarded as the initiator of HDL-C reduction [27]. On the other hand, some key enzymes involved in HDL metabolism, such as CETP, LCAT, hepatic lipase(HL) and protein phospholipid transfer protein(PLTP), are changed in obese people with insulin resistance, promoting this process [28]. What's more, the increased plasma clearance of apoA-I and downregulation of its production also offer some contributions to the reduction of HDL-C [29]. In spite of these interpretations, there is no consolidated explanation for the association between obesity and decreased HDL-C levels. In the following part, we will review several recent findings that provide new clues to help us understand the potential mechanism underlying obesity and HDL degeneration.

## Changes in HDL component

### HDL clearance accelerates when it gets fat

HDL-C level reflects the balance between the rate of HDL clearance and the rate of HDL production. However, studies have demonstrated that increased clearance of HDL undertook more responsibilities for HDL decrease in obese individuals [30]. And further studies showed the compositional changes in HDL, especially triglyceride enrichment, accounted for the majority of causes of its clearance enhancement. Under the states of obesity and insulin resistance, adipose tissue releases more free fatty acids (FFAs), the substrate for VLDL formation in liver, to circulation. The elevated VLDL levels in plasma will lead more TG in VLDL to be transferred to HDL through the act of CETP [31]. Moreover, CETP activity increases significantly in obese subjects [32]. As a result, a greater number of HDL particles are cholesterol depleted and TG enriched. Hepatic lipase (HL), which is also increased in obesity [33], hydrolyzes TG-rich HDL, releasing lipid-poor apoA-I and forming remnant HDL particles(α-migrating, lipolytically modified HDLs) [34]. Lipid-poor apoA-I can be recycled to form nascent HDL particles, but more likely to be cleared by kidney. On the other hand, HDL remnants bind to liver or kidney that mediates the remnants uptake, internalization, and degradation.

However, there is no agreed opinion concerning the mechanisms of enhanced HDL remnant clearance. One explanation proposed by Xiao et al [35] demonstrated that compared to native and TG-rich HDL, the binding of remnant HDL was markedly higher in both HepG2 cells and HEK293 cells. Remnant HDL was internalized to a greater extent in both cell types and was more readily degraded in HEK293 cells. The authors also demonstrated that the increased binding of HDL remnant was not mediated by the low-density lipoprotein receptor (LDLR) or SR-BI. In addition, Brown et al [36] suggested that endothelial lipase (EL), which is also elevated in obesity and insulin resistance [37], hydrolyzed phospholipids from remnant HDL particles and further enhanced their catabolism. However, the molecular pathways of uptake and degradation of HDL remnants require further studies.

And so far, there is still no knowing if the TG-rich HDL (before or after hydrolysis by HL) has decreased function of cholesterol efflux and RCT. Previous study has shown that in obese individuals, the conversion of pre-β1 to pre-β2 HDL was inhibited [38]. The pre-β1 HDL (similar to nascent HDL) is initial mediator of cholesterol efflux from peripheral cells. But the increase of pre-β1 HDL in obesity did not result in enhancement of transfer of cholesterol from peripheral cells to HDL subfractions. On the contrary, the peripheral cholesterol transfer to HDL subfractions was impaired in obesity. These results suggest that the changes in HDL components would have some effects on its functions.

### Prevent HDL from getting fat

As mentioned above, in the process of triglycerides transferring from VLDL to HDL, CETP plays an indispensable role, especially in obese and insulin resistant states. Humans genetically deficient in CETP had markedly elevated HDL-C [39]. Animal experiments revealed that inhibitions of CETP blocked the exchange of TG and cholesterol, thus prevented HDL from getting fat and effectively raised HDL-C levels [40]. Hence, inhibition of CETP with CETP inhibitors, such as torcetrapib, was believed to be beneficial for HDL-C metabolism and finally prevent cardiovascular events [41]. Unfortunately, the large clinical trial ILLUMINATE was stopped prematurely as a result of an excess of deaths and morbidity in the group receiving torcetrapib and atorvastatin compared to atorvastatin alone [42]. However, further studies suggested that the HDL from patients treated with torcetrapib was functional and promoted regression of atherosclerosis and the failure may have been caused by off-target toxicity of torcetrapib [42]. Despite the failure of torcetrapib, inhibition of CETP still seems to be a sound strategy for increasing HDL-C. Recent clinical trial has demonstrated that anacetrapib (another CETP inhibitor) had robust impact on LDL and HDL cholesterol and did not result in the adverse effects observed with torcetrapib [43].

Besides CETP, the factors that associated with hypertriglyceridemia are also potential targets of preventing the changes in HDL component for the reason that hypertriglyceridemia is an initial requirement for the overproduction of TG-rich HDL. Chan et al [44] showed that elevated plasma apoC-III was a predictor of hypercatabolism of HDL and apoA-I. By inhibiting lipoprotein lipase and hepatic uptake, apoC-III can impair the hydrolysis of TG [45]. The elevated level of apoC-III in plasma, which was associated with insulin resistance, will promote the formation of TG-rich HDL. Moreover, adipose fatty acid binding protein 4 (FABP4) which is also elevated in obesity has a significant positive correlation with TG-rich VLDL and inverse correlation with HDL-C. And these correlations are not affected by age, gender, BMI or other factors, indicating that FABP4 may directly modulate HDL-C metabolism [46]. Other researches found that the accumulation of fat in liver obviously increased the production of TG-rich HDL [47,48]. Although further studies are still needed, reducing the levels of apoC-III, FABP4 and liver fat would prevent the changes in HDL component and help to ameliorate HDL-C metabolism, especially in obesity and insulin resistance [49].

## Effect of hypertrophied adipocyte on HDL

### Efflux of cholesterol from hypertrophied adipocyte decreases

Adipose tissue is the biggest tissue in body which can store large amounts of lipids, and therefore it is potentially a reservoir of cholesterol. Thus the impact of adipose tissue on HDL metabolism is possible immense, especially in obesity. Adipocytes are the predominant cells in adipose tissue and their main function is to store excess energy as the form of TG. Additionally, adipose tissue is also recognized as an endocrine organ because adipocytes have the ability to secrete a large number of cytokine which named as adipokines, such as leptin, adiponectin, tumor necrosis factor-α (TNF-α) and interleukin-1β (IL-1β) [50]. In obesity, abnormal excess energy leads to adipocytes obviously enlarge and the hypertrophied adipocytes manifest several altered metabolic properties that play a key part in the obesity related dyslipidemia.

As previously mentioned, cholesterol efflux to HDL, especially from macrophage, is the main antiatherogenic effect of HDL. However, the cholesterol efflux from macrophage and its expression of ABCA1 has minimal contribution to plasma HDL-C levels [51]. In contrast, adipose tissue contains the largest pool of free cholesterol and in obese adults it contains over 50% of the total body cholesterol [52,53]. Hence, for obese individuals, cholesterol efflux from this peripheral tissue may play a relatively important role in modulation of the whole circulating HDL-C levels. A research group injected 3T3-L1 adipocytes which labeled with ^3^H-cholesterol into the bodies of mice and tracked movement of label onto plasma HDL [54]. The authors demonstrated that adipocytes supported transfer of cholesterol to HDL in vivo as well as in vitro and provided evidence that adipocyte cholesterol efflux was controlled by ABCA1 and SR-BI, but not ABCG1. Furthermore, TNF-α, an inflammatory factor, reduced both ABCA1 and SR-BI expression and impaired cholesterol efflux from adipocytes. These results indicated that adipocyte inflammation impaired its cholesterol efflux to HDL. Thus, as adipose inflammation is a hallmark of obesity and insulin resistance [50], loss of adipocyte cholesterol efflux to HDL may directly contribute to low HDL-C levels related to obesity.

Another group found that activation of the lipolysis of 3T3-L1 adipocytes promoted a 22% increase in cholesterol efflux to HDL particles [55]. However, the enhancement of cholesterol efflux was not due to the levels of ABCA1 and SR-BI. Brefeldin A-sensitive vesicular transport contributed to the major part in the enhancement of cholesterol efflux associated with adipocytes lipolysis. In consistent with this, sustained weight loss through dieting or exercise which leads to fat mobilization of adipocytes could reverse the decrease of HDL-C levels in obesity [56,57].

### Influx of cholesterol into hypertrophied adipocyte increases

Although adipose tissue contains large amounts of cholesterol, the capacity of cholesterol synthesis of adipocytes is extremely limited [58]. The cholesterol accumulation of adipose tissue is dependent on the influx of cholesterol. Adipocytes can selectively uptake CE from HDL through both SR-BI dependent and SR-BI independent pathways [59]. In both of the uptake pathways, the CE cargo of HDL is selectively unloaded into cells without internalization and degradation of the entire lipoprotein [60]. However, several researches observed that with the increased uptake of CE form HDL, plasma HDL-C levels were rapidly reduced [61,62]. Our recent experiments have shown that the adipose tissue from greater omentum of human retained a large amount of apoA-I (unpublished data). Hence, we speculated that the increased cholesterol influx into adipocytes is accompanied by the internalization of apoAI as no de novo synthesis of apoAI in adipocyte was noted.

### Adipokines affect HDL metabolism

In the state of obesity, the secretion of adipokines is also altered. Nearly all of the adipokines are increased in obesity except for adiponectin. Adiponectin (APN) is a peptide mainly synthesized in adipose tissue and plays an important role in modulating glucose metabolism and inhibiting atherosclerosis [63]. A great number of studies have shown that APN levels were reduced in individuals with obesity and that weight loss could increase APN levels [64,65]. As an antiatherogenic adipokine, APN has critical influence on HDL metabolism. Human studies have found that plasma APN concentrations were positively correlated with HDL-C levels and the relationship is independent of BMI, body fat distribution and insulin sensitivity [66,67]. Verges et al verified that APN was a critical determinant of apoA-I catabolism. There was a significant negative correlation between plasma APN and apoA-I FCR in patients with metabolic syndrome and normal subjects [68]. In vitro experiments demonstrated that APN enhanced mRNA expression and protein secretion of apoA-I from HepG2 cells [69]. Furthermore, APN could upregulate ABCA1 at both mRNA and protein levels in HepG2 cells, suggesting that APN might increase HDL assembly through enhancing ABCA1 pathway and apoA-1 synthesis in the liver. In consistent with that, the apoA-I protein levels and ABCA1 expression in liver were reduced in APN-knockout mice compared with wild-type mice [70]. What's more, APN could reduce the release of ApoB and ApoE from hepatocytes, resulting in reduced release of TG-rich lipoproteins from the liver thus preventing the formation of TG-rich HDL and leading to elevated systemic HDL-C [71].

In obese individuals, the inflammatory cytokines which released from hypertrophied adipocytes are raised and may be directly associated with the reduction of HDL-C and CVD development. The pro-inflammatory state is a main feature of obesity. In other inflammatory states such as rheumatoid arthritis or acute infections, plasma HDL-C levels are also obviously reduced [72,73]. TNF-α and IL-1β, as universal inflammatory cytokines, have been identified to downregulate apoA-I gene expression in hepatocytes with a dose-dependent manner [74]. Further studies revealed that TNF-α suppressed apoA-I promoter activity through both the MEK/ERK and JNK pathways [75].

**Figure 1:**
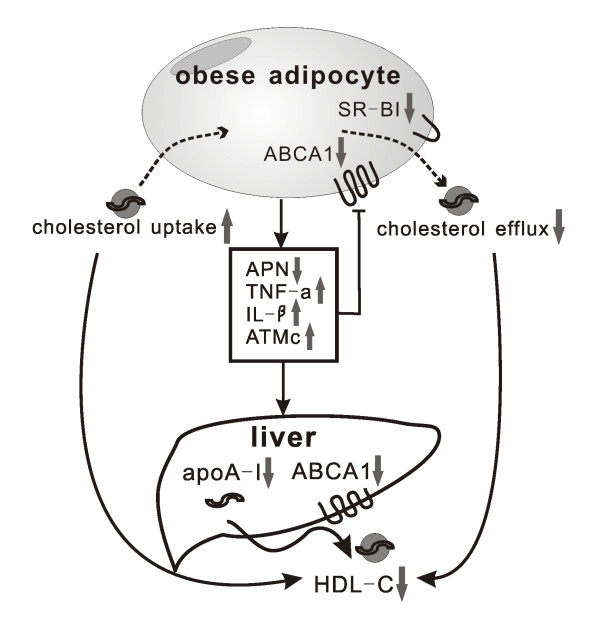
**Effect of adipocyte on HDL**. Hypertrophied adipocytes manifest several altered metabolic properties that play a role in lowered HDL-C. Adipocyte inflammation impairs its cholesterol efflux to HDL while cholesterol influx to adipocyte is increased in the state of obesity. In addition, altered adipokines downregulate apoA-I and ABCA1 gene expression in hepatocytes, thus reduce HDL assembly in the liver. ABCA1, ATP-binding cassette transporter A1; SR-BI, scavenger receptor type-BI; APN, adiponectin; TNF-a, tumor necrosis factor-α; IL-1β, interleukin-β; ATMc, adipose tissue macrophage content; apoA-I, apolipoprotein AI; HDL-C, high-density lipoprotein cholesterol.

Besides adipocytes, adipose tissue macrophage content (ATMc) is also associated with increasing adiposity. Moreover, ATMc is another determinant for lower HDL-C level independent of BMI [24]. This suggests that ATMc also have influence on the HDL metabolism. In addition, in vivo experiments provided evidence that inflammation impaired RCT at multiple steps in its pathway. Impairment of RCT and HDL efflux function, independent of HDL-C levels, may contribute to atherosclerosis in chronic inflammatory states including obesity and diabetes [76]. In vitro experiments displayed similar results that inflammatory cytokines down regulated the expression of ABCA1 and ABCG1 in mouse macrophages [77], thus blocked cholesterol efflux from macrophages and promoted atherosclerosis formation. (Figure 1).

## Effect of hepatocytes on HDL

### Hepatic ABCA1 is key to circulating HDL-C

Liver is the centric organ for lipid metabolism including LDL, TG, and HDL. Although hepatic apoA-I is the major protein source of HDL, hepatic ABCA1 seems indispensable to the production of circulating HDL. Animal study has revealed that overexpression of adenovirally delivered ABCA1 in the liver of mice resulted in significant increase in plasma HDL-C [78]. Similarly, transgenic mice overexpressing ABCA1 in the liver showed prominent elevation of plasma HDL-C [79]. On the other hand, 80% decrease in plasma HDL-C was observed in mice with liver-specific deletion of ABCA1 [80]. These studies suggested that hepatic ABCA1 is critical in maintaining the circulating HDL-C levels by formation of nascent HDL particles [81]. Although extrahepatic ABCA1 is essential for the maturation of HDL particles, it contributes little to the plasma HDL-C levels [82,83]. However, the regulation of hepatic ABCA1 in obesity and insulin resistance has not yet been clearly defined.

### Hepatic miR-33 as novel regulatory pathway of HDL-C

In the search of the regulatory factors of intracellular cholesterol homeostasis, two independent groups have concurrently discovered a novel pathway----microRNA-33a(miR-33a) and its host gene SERBP-2 [84,85]. SREBP-2(sterol regulatory element-binding protein-2) has been recognized as an important sensor of intracellular cholesterol. It can control hepatic cholesterol homeostasis by regulating cholesterol synthesis via HMG-CoA reductase and cholesterol uptake via LDL-receptor. MiR-33a is located in the intron of SREBP-2 gene and appears to be co-regulated with the SREBP-2 by cellular cholesterol content.

MiR-33a was identified to target ABCA1, resulting in ABCA1 silencing through post-transcriptional repression. As a consequence of this targeting, miR-33a limited the efflux of cholesterol to apoA-I in both macrophages and hepatocytes. Overexpression of miR-33 in mice resulted in significantly reduced hepatic ABCA1 expression as well as a progressive decline of plasma HDL-C. Conversely, mice expressing anti-miR-33 showed a 50% increase in hepatic ABCA1 protein and a concomitant 25% increase in plasma HDL-C levels [84]. Horie et al [86] generated miR-33-deficient mice and revealed that genetic deletion of miR-33 resulted in increased hepatic ABCA1 expression and elevated apoA-I dependent cholesterol efflux. Moreover, miR-33-deficient mice had significant higher serum HDL-C levels than WT mice (25%). Rayner et al [87] studied the impact of miR-33 inhibition in mice deficient for LDL receptor (Ldlr^-/- ^mice), with established atherosclerotic plaques. Ldlr^-/- ^mice treated with anti-miR-33 showed an increase in plasma HDL-C levels and a reduction in plaque size with an increase in plaque stability. These studies suggest that raising HDL-C levels by anti-miR-33 treatment promotes atherosclerosis regression and holds tremendous potential for the prevention and treatment of CVD.

Notably, in addition to the cholesterol transport genes, miR-33 also inhibits translation of several transcripts encoding proteins involved in fatty acid β-oxidation, including CPT1A(carnitine palmitoyltransferase 1A), CROT(carnitine O-octanoyltransferase) and HADHB(β-subunit of the mitochondrial trifunctional protein) [88]. Each of them plays a indispensable role in the fatty acid oxidation pathway. Thereby, overexpression of miR-33a led to reduced fatty acid degradation and lipid accumulation in liver. Thus, the transcript encoding SREBP-2 and miR-33a contains a protein that increase lipid synthesis and a microRNA that prevent export and degradation of newly synthesized lipids.

### Hepatic miR-33b and SREBP-1c may coordinate the HDL-C and TG variation in obesity

Besides miR-33a, another member of the miR-33 family, miR-33b, is found within the intron of human SREBP-1c gene. There are only two nucleotides difference between miR-33a and miR-33b. However, the two-nucleotide variation does not appreciably affect the gene targeting by these miRNAs [89]. As for the host gene SREBP-1c, it is the predominate isoform of SREBPs in the liver and preferentially enhances transcription of the genes involved in fatty acid synthesis but not cholesterol synthesis [90]. In addition, the transcriptional regulation of the SREBP-1c is complex and is of great importance for the metabolism homeostasis of fatty acid. Insulin was shown to be a key regulator of SREBP-1c gene expression [91,92]. Insulin selectively stimulates SREBP-1c transcription in liver and elevated SREBP-1c in turn mediates insulin-stimulated fatty acid synthesis. Further researches revealed that the alternation of adipokines also had impacts on SREBP-1c expression. TNF-α could induce hepatic SREBP-1c expression [93] and adiponectin could suppress hepatic SREBP-1c expression [94].

As we know, obesity has a strong association with insulin resistance, hyperinsulinemia and disorders of adipokines. In consistent with that, upregulation of SREBP-1c in liver was observed in both obese patients [95,96] and mouse models [97]. As mentioned above, miR-33b is encoded in human SREBP-1c gene. Furthermore, increased expression of miR-33b was observed when activating SREBP-1 with an LXR agonist [89]. It is reasonable to deduce that the expression of miR-33b in liver would be activated in obesity with the upregulation of SREBP-1c gene.

**Figure 2:**
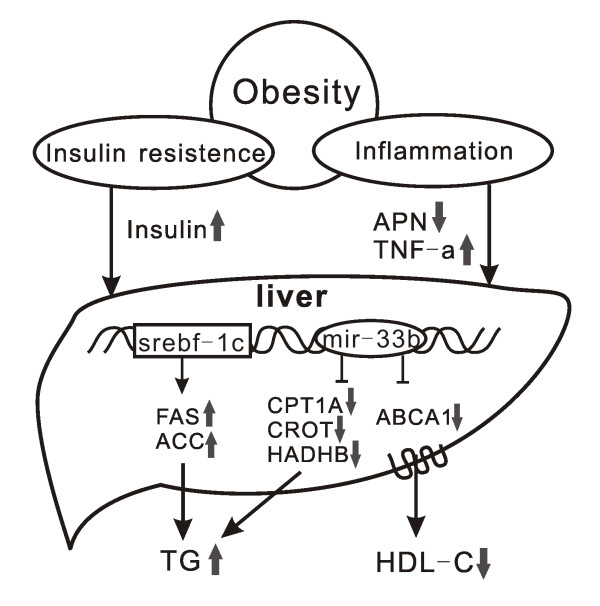
**Hepatic miR-33 and its host gene coordinate HDL-C and TG variations in obesity**. In the state of obesity, hyperinsulinemia and disorders of adipokines would induce the expression of hepatic SREBP-1c gene as well as miR-33b. The upregulation of SREBP-1c would active its downstream genes and accelerate TG synthesis; upregulation of miR-33b would repress hepatic HDL assembly through the inhibition of ABCA1 and promote fat accumulation through the repression of fatty acid degradation. APN, adiponectin; TNF-a, tumor necrosis factor-α; srebf-1c, sterol regulatory element-binding transcription factor; miR-33b, microRNA-33b; FAS, fatty acid synthetase; ACC, acetyl-CoA carboxylase; CPT1A, carnitine palmitoyltransferase 1A; CROT, carnitine O-octanoyltransferase; HADHB, β-subunit of the mitochondrial trifunctional protein; ABCA1, ATP-binding cassette transporter A1; HDL-C, high-density lipoprotein cholesterol; TG, triglyceride.

Additionally, our primary data showed that compared to the solo-cultured hepatocytes, hepatic cells co-cultured with adipocytes presented increased expression of SREBP-1c while decreased expression of ABCA1. This indicated that the paracrine of adipocytes had effects on hepatic lipid metabolism, both on TG and HDL. However, the specific links between liver and adipose tissue have not yet been identified. According to the researches we mentioned above, we propose that in the state of obesity, hyperinsulinemia and disorders of adipokines would induce the expression of hepatic SREBP-1c gene as well as miR-33b. The upregulation of SREBP-1c would activate its downstream genes, such as fatty acid synthetase (FAS) and acetyl-CoA carboxylase (ACC) and accelerate TG and VLDL synthesis. At the same time, upregulation of miR-33b would repress hepatic HDL assembly through the inhibition of ABCA1 and promote fat accumulation through the repression of fatty acid degradation. Thus, elevated plasma TG level and reduced HDL-C level would be manifested simultaneously in obese individuals (Figure 2).

Interestingly, although miR-33a is highly conserved in mammals, miR-33b is different between humans and mice. SREBP-1c gene in human encodes miR-33b, but there is no miR-33b in mice. This is consistent with the fact that obese and insulin-resistant mouse models manifest all of the human features of metabolic disorders except a reduction in HDL-C. Conversely, in obese mice, the high level of cholesterol would suppress SREBP-2 and miR-33a expression. This is consistent with the observation that dietary fat induced obesity slightly increases HDL-C levels in mice [98].

## Conclusions

As a conclusion, low level of HDL-C is a common lipid disorder in obese people and plays an indispensible role in the development of CVD. Both the factors intrinsic to HDL particles and extrinsic to HDL particles are involved in the abnormal HDL metabolism in obesity. In obese individuals, the changes in HDL component play a major role in its rapid clearance. Both cholesterol efflux and influx of adipocytes may affect plasma HDL-C levels. And the alternations of adipokines in obesity also contributed to low HDL-C. In addition, we propose that hepatic miR-33b and its host gene SREBP-1c coordinate HDL-C and TG variations. These new findings provide new clues to the understanding of the potential mechanism of low HDL-C in obesity and potential targets for preventing and treating HDL-C reduction.

## Conflict of interests

The authors declare that they have no competing interests.

## Authors' contributions

HW and DQP conceived the study, its design and drafted the manuscript. All authors read and approved the final manuscript.
